# Gut Microbiome and Its Role in Parkinson's Disease

**DOI:** 10.7759/cureus.73150

**Published:** 2024-11-06

**Authors:** Suchith B Suresh, Aparna Malireddi, Mahlet Abera, Khutaija Noor, Mehwish Ansar, Sruthi Boddeti, Tuheen Sankar Nath

**Affiliations:** 1 Internal Medicine, Montefiore St. Luke's Cornwall, Newburgh, USA; 2 Internal Medicine, Andhra Medical College, Visakhapatnam, IND; 3 Internal Medicine, Saint Paul's Hospital Millennium Medical College, Addis Ababa, ETH; 4 Foundation of Clinical Research, Harvard Medical School, Boston, USA; 5 Neuropsychiatry, PsychCare Consultants Research, St. Louis, USA; 6 Internal Medicine, Shadan Institute of Medical Sciences, Hyderabad, IND; 7 General Surgery, Wirral University Teaching Hospital, Wirral, GBR; 8 Internal Medicine, Tirumala Jyoti Hospital, Anakapalle, IND; 9 Surgical Oncology, Tata Medical Center, Kolkata, IND

**Keywords:** alpha-synuclein, fecal microbiota transplant, gut dysbiosis and diseases, gut microbiomes, parkinson's disease

## Abstract

Parkinson's disease (PD) afflicted more than 8.5 million people globally in 2019, as the prevalence of the condition more than doubled during the preceding 25 years. Both non-motor symptoms, such as mood disorders and cognitive impairment, and motor symptoms, such as tremors and rigidity, are indicative of this progressive neurodegenerative disease. Recent data indicates a significant role for the gut microbiome in PD pathogenesis and progression, emphasizing the microbiota-gut-brain axis. In compliance with the Preferred Reporting Items for Systematic Reviews and Meta-Analyses (PRISMA) 2020 statement, this systematic review summarizes our current knowledge about the function of the gut microbiome in PD, highlighting recurrent microbial alterations and assessing microbiome-based treatment strategies. The review revealed several consistent patterns in the gut microbiota of PD patients, including reduced microbial diversity and specific taxonomic alterations, including a drop in *Firmicutes* abundance and an increase in *Proteobacteria* abundance. Functional changes in the gut microbiome, such as altered short-chain fatty acid (SCFA) production and tryptophan metabolism, were also noted. These microbial changes were observed even in early-stage and drug-naïve PD patients, suggesting they are not merely a consequence of disease progression or medication use. The review highlighted potential mechanisms linking gut microbiome alterations to PD, including increased intestinal permeability, neuroinflammation, and modulation of alpha-synuclein aggregation. Probiotics, prebiotics, and fecal microbiota transplantation are a few interventions that try to modify the gut microbiome and might be possible to halt the advancement of PD and enhance patients' quality of life with the condition. Future research should focus on establishing causality through large-scale longitudinal studies, standardizing microbiome analysis methods, and exploring personalized microbiome-based therapies.

## Introduction and background

In the last 25 years, the prevalence of Parkinson's disease (PD) has more than doubled. The World Health Organization (WHO) estimated that 8.5 million people worldwide had PD in 2019. Moreover, PD is associated with a significant increase in disability and mortality rates. In 2019, PD caused 329,000 fatalities and 5.8 million disability-adjusted life years (DALYs), representing an increase of 81% and more than 100%, respectively, since 2000 [1].

PD is an advancing neurological illness that occurs as both non-motor and motor symptoms. Tremors, rigidity, and bradykinesia are examples of motor symptoms; mood disorders, sleep problems, cognitive impairment, and autonomic dysfunction are some of the non-motor symptoms. Alpha-synuclein aggregation and dopaminergic neuron loss in the substantia nigra are long associated with PD. However, new research emphasizes the significance of the microbiota-gut-brain axis and points to a significant role of the gut microbiome in the pathophysiology and progression of PD [1].

Recent research has repeatedly shown that the gut microbiota of people with PD differs significantly from that of healthy people. Key findings include a decreased abundance of beneficial bacteria such as *Prevotellaceae*, *Lachnospiraceae*, and short-chain fatty acid (SCFA)-producing bacteria and an increased presence of potentially harmful bacteria like *Verrucomicrobiaceae* and *Akkermansia*. These microorganism changes have been linked to disease progression and symptom severity.

The changes in microbial composition are believed to contribute to the pathogenesis of PD through several mechanisms. These mechanisms include promoting alpha-synuclein aggregation, inducing intestinal and systemic inflammation, disrupting gut barrier integrity, and modulating neuroinflammation and neurodegeneration. Furthermore, the observation that gastrointestinal symptoms often precede motor symptoms by several years suggests a potential early role in disease onset, further supporting the gut microbiome's influence on PD.

Ongoing research focuses on demonstrating specific microbial changes associated with PD and understanding the underlying mechanisms by which these changes contribute to disease progression. Investigators are also exploring the capacity of gut microbiota as a diagnostic biomarker for early PD detection and as a therapeutic target. Furthermore, preliminary studies are exploring the potential of microbiome-targeted therapies, such as fecal microbiota transplant (FMT), prebiotics, probiotics, and dietary changes, in altering the gut microbiome and their possible effects on PD symptoms. While these investigations show promise, it's important to note that they are still in the early stages and definitive conclusions about their efficacy in lessening PD symptoms cannot yet be drawn. Large-scale studies are underway to characterize further gut microbiome alterations in PD and their relationship to clinical features. These studies also aim to examine microbial metabolites' role in PD pathophysiology, particularly SCFAs. However, the complex interplay between the gut microbiome and PD requires extensive further research before any clinical applications can be confidently recommended.

Despite these advancements, several knowledge gaps still need to be addressed. While causality is debated, recent evidence points to a possible bidirectional relationship between gut microbiome changes and PD. There is considerable variability in microbial findings across different studies, probably due to the research populations' variations, methodologies, and environmental factors. The specific mechanisms by which gut bacteria and their metabolites influence neurodegeneration in PD have yet to be understood entirely. Furthermore, the long-term effects and optimal protocols for microbiome-targeted interventions in PD management are yet to be determined, and the impact of individual variations in microbiome composition on PD risk, progression, and treatment response requires further exploration.

This systematic study aims to summarize the current understanding of the gut microbiome's function in PD, identify consistent microbial changes and underlying mechanisms across studies, evaluate the potential of microbiome-based therapeutic approaches, provide a comprehensive understanding of the gut-brain connection in PD, and highlight future research directions to fill existing knowledge gaps.

## Review

Methods

Following the Preferred Reporting Items for Systematic Reviews and Meta-Analyses (PRISMA) 2020 declaration, this systematic review was carried out and reported. Table 1 shows the summary of the included studies.

**Table 1:** Summary of the included studies PD: Parkinson's disease; SCFA: short-chain fatty acid; *H. pylori*: *Helicobacter pylori*; CRP: C-reactive protein; UPDRS: Unified Parkinson's Disease Rating Scale; CNS: central nervous system; ENS: enteral nervous system; FMT: fecal microbiota transplant; VLDL; very-low-density lipoprotein; HDL: high-density lipoprotein References: Omotosho et al. [2], Huang et al. [3], Çamcı and Oğuz [4], Keshavarzian et al. [5], Menozzi et al. [6], Pavan et al. [7], Forsyth et al. [8], Fitzgerald et al. [9], Lei et al. [10], Aho et al. [11], Liu et al. [12], Fan et al. [13], Tamtaji et al. [14], Zhu et al. [15], Hegelmaier et al. [16], Menozzi and Schapira [17], Cheng et al. [18], Kang et al. [19], Lorente-Picón and Laguna [20], Jackson et al. [21]

Author(s)	Year	Type of study	Number of patients	Purpose of study	Key findings
Omotosho et al.	2023	Review	NA	Gut dysbiosis on PD	Gut microbiota composition impacts PD progression and pathogenesis; gastrointestinal symptoms often precede motor symptoms in PD and Braak's hypothesis
Huang et al.	2021	Review	NA	Gut dysbiosis on PD	Gut dysbiosis contributes to PD through inflammation, oxidative stress, and barrier disruption. Decreased butyrate-producing bacteria, *H. pylori* infection potentially linked to PD, increased *Akkermansia muciniphila* associated with PD
Çamcı and Oğuz	2016	Review of observational studies	358 PD	Association between *H. pylori* and PD	Higher prevalence of *H. pylori* in PD patients; eradication improves levodopa treatment; *H. pylori* infection can impact the course of PD
Keshavarzian et al.	2016	Case-control study	72 (38 PD patients, 34 healthy controls)	To characterize the colonic bacterial composition in PD patients compared to healthy controls	Significant differences in mucosal and fecal microbial communities between PD patients and controls. Anti-inflammatory bacteria were more abundant in controls, while pro-inflammatory bacteria were more abundant in PD patients. PD fecal microbiome showed a lower abundance of genes involved in metabolism and a higher abundance of genes involved in lipopolysaccharide biosynthesis and type III bacterial secretion systems
Menozzi et al.	2021	Review	-	Focuses on the gut-brain axis hypothesis and its clinical implications	Evidence supports PD potentially originating in the gut. Two PD subtypes were proposed: gut-first and brain-first. Gastrointestinal disorders impact PD patients' lives and may indicate early disease
Pavan et al.	2022	Review	-	Potential of gut microbial signatures as predictors and early diagnostic markers for PD	Pathophysiology of inflammation due to gut dysbiosis. Higher levels of calprotectin, alpha-1-antitrypsin, and zonulin in the feces of PD patients
Forsyth et al.	2011	Cross-sectional study	9 PD vs 10 controls	Gut dysbiosis leading to inflammation	Increased markers of intestinal permeability
Fitzgerald et al.	2019	Review	-	Pathogenesis of alpha-synucleinopathies	Alpha-synuclein folding and aggregation are modulated by gut dysbiosis; vagotomy lowers the risk of PD
Lei et al.	2021	Review	-	Discuss alpha-synuclein's role in gut microbiome-related PD progression	Gut microbiome changes lead to alpha-synuclein misfolding and PD progression
Aho et al.	2021	Cross-sectional study	55 PD patients, 56 controls	Investigate gut microbiota, SCFAs, inflammation, and gut permeability in PD	Lower SCFA levels; higher inflammation markers in PD patients
Liu et al.	2024	Review	-	Investigate SCFA's role in PD pathology	Altered SCFA levels in PD; potential therapeutic effects of SCFAs
Fan et al.	2022	Review	-	Summarize gut microbiota-PD connection and therapeutic targets	Specific bacterial changes in PD; roles of gut-brain communication pathways
Tamtaji et al.	2020	Meta-analysis	-	Effects of probiotic supplementation on metabolic profiles in patients with neurological disorders	Probiotic supplementation significantly reduced CRP, insulin, triglycerides, and VLDL cholesterol levels while increasing HDL cholesterol levels
Zhu et al.	2022	Review	-	Microbiota-targeted therapeutic approaches in PD	Improvement in motor symptoms, sleep quality, constipation, and cognition with probiotics
Hegelmaier et al.	2020	Case-control study	54 PD patients, 32 healthy controls	To assess the impact of dietary intervention and bowel cleansing on gut microbiome and motor symptoms in PD	UPDRS III scores improved after a vegetarian diet and fecal enema. Levodopa-equivalent daily dose decreased. Gut microbiome diversity associated with UPDRS III scores. Abundance of *Clostridiaceae *reduced after enema
Menozzi and Schapira	2024	Review	-	Overview of gut microbiota-PD interactions and medication effectiveness	Gut microbiota influences levodopa absorption and side effects
Cheng et al.	2023	RCT	56 PD patients (27 FMT, 27 placebo)	Efficacy of FMT in PD	FMT improved PD-related autonomic symptoms compared to placebo; FMT improved gastrointestinal disorders
Kang et al.	2021	Review	-	To examine the association between gut microbiota and PD and prospects of FMT treatment	Gut microbial dysbiosis affects both CNS and ENS in PD. Some gut microbes may suppress neuroinflammation and gut inflammation. FMT may serve as a potential treatment for PD in the future
Lorente-Picón and Laguna	2021	Review	-	To review microbiota-based therapeutic strategies for PD	Discusses antibiotics, probiotics, prebiotics, synbiotics, dietary interventions, FMT, and live biotherapeutic products as potential strategies
Jackson et al.	2019	Review	-	To review the role of diet and microbiome in PD	The Western diet is associated with increased PD risk, while the Mediterranean diet is associated with decreased PD risk

Search Strategy

A comprehensive literature search was conducted across three electronic databases: the Cochrane Library, PubMed, and Google Scholar. The search strategy employed is described in Table 2.

**Table 2:** Search strategy

Database	Search strategy	Results
PubMed	Gut microbiome or gut dysbiosis(( "Gastrointestinal Microbiome/drug effects"[Majr] OR "Gastrointestinal Microbiome/genetics"[Majr] OR "Gastrointestinal Microbiome/immunology"[Majr] OR "Gastrointestinal Microbiome/physiology"[Majr] )) AND Parkinson disease ( "Parkinson Disease/diet therapy"[Majr] OR "Parkinson Disease/drug therapy"[Majr] OR "Parkinson Disease/etiology"[Majr] OR "Parkinson Disease/pathology"[Majr] OR "Parkinson Disease/physiopathology"[Majr] OR "Parkinson Disease/prevention and control"[Majr] OR "Parkinson Disease/therapy"[Majr] )	108
Cochrane Library	[Gastrointestinal Microbiome] and [Parkinson Disease]	8
Google Scholar	gut microbiome and its role in parkinson disease trials	35400

Eligibility Criteria

The inclusion and exclusion criteria are included in Table 3.

**Table 3:** Eligibility criteria

Inclusion	Exclusion
<5 years	>5 years
Human	Animal
Full text	Not full text
English	Other languages
Only Parkinson	Other neurodegenerative diseases
	Irrelevant outcome

Study Selection

Two separate reviewers initially screened the titles and abstracts of all retrieved publications to compare them with the eligibility criteria. This was the first stage of the two-stage study selection process. Any disagreements were resolved through discussions or by seeking advice from three reviewers. Then, the second set of reviewers independently extracted and evaluated all the texts of the potentially relevant papers during a full-text review. In case of disagreements, they were resolved through arbitration or by reaching a consensus among the three reviewers. One hundred and eight articles matched the original search query. After removing duplicates and applying the inclusion and exclusion criteria, 41 articles were still pending full-text evaluation. After careful consideration, a final group of 23 papers was selected for the systematic review.

Quality Assessment

The Cochrane risk-of-bias tool for randomized controlled trials (RCTs) (Table 7), the Newcastle-Ottawa Scale for observational studies (Table 5 and Table 6) for observational studies, AMSTAR 2 for meta-analysis (Table 8), and the SANRA checklist (Table 4) for review articles were used to evaluate the methodological quality of the included papers.

**Table 4:** SANRA checklist References: Omotosho et al. [2], Huang et al. [3], Çamcı and Oğuz [4], Menozzi et al. [6], Pavan et al. [7], Fitzgerald et al. [9], Lei et al. [10], Liu et al. [12], Fan et al. [13], Tamtaji et al. [14], Zhu et al. [15], Menozzi and Schapira [17], Kang et al. [19], Lorente-Picón and Laguna [20], Jackson et al. [21], Liang et al. [22]

Selection	Justification of the article's importance for the readership	Statement of concrete aims or formulation of questions	Description of the literature search	Referencing	Scientific reasoning	Appropriate presentation of data	Total
Omotosho et al.	2	2	1	2	2	1	10
Huang et al.	2	2	0	2	2	2	10
Çamcı and Oğuz	2	1	0	2	2	2	9
Menozzi et al.	2	2	2	2	2	2	12
Pavan et al.	2	2	1	2	2	2	11
Fitzgerald et al.	2	2	1	2	2	2	11
Lei et al.	2	2	0	2	2	2	10
Liu et al.	2	2	2	2	2	2	12
Fan et al.	2	2	1	2	2	2	11
Tamtaji et al.	2	2	2	2	2	2	12
Zhu et al.	2	2	1	2	2	2	11
Menozzi and Schapira	2	2	0	2	2	2	10
Kang et al.	2	2	1	2	2	2	11
Lorente-Picón and Laguna	2	2	0	2	2	2	10
Jackson et al.	2	2	1	2	2	2	11
Liang et al.	2	2	0	2	2	1	9

**Table 5:** The Newcastle-Ottawa tool for case-control studies References: Keshavarzian et al. [5], Hegelmaier et al. [16]

Case-control studies	Keshavarzian et al.	Hegelmaier et al.
Is the case definition adequate?	1	1
Representativeness of the cases	0	1
Selection of controls	1	0
Definition of controls	1	2
Comparability of cases and controls on the basis of the design or analysis	1	2
Ascertainment of exposure	1	2
The same method of ascertainment for cases and controls	1	1
Non-response rate	0	0
Total	7	9

**Table 6:** The Newcastle-Ottawa Scale for cross-sectional studies References: Aho et al. [11], Forsyth et al. [8]

Cross-sectional studies	Aho et al.	Forsyth et al.
Representativeness of the sample	1	1
Sample size	1	0
Non-respondents	0	0
Ascertainment of exposure	2	2
Comparability	2	1
Assessment of outcome	2	2
Statistical test	1	1
Total	9	7

**Table 7:** Cochrane risk-of-bias assessment tool References: Cheng et al. [18], Sathe et al. [23]

Randomized controlled trial	Cheng et al.	Sathe et al.
Bias arising from the randomization process	Some concerns	Not applicable
Bias due to deviations from intended interventions	Low risk	Some concerns
Bias due to missing outcome data	Low risk	Low risk
Bias in the measurement of the outcome	Some concerns	Some concerns
Bias in the selection of the reported result	Low risk	Low risk
Final	Included	Included

**Table 8:** AMSTAR 2 Reference: Iftikhar et al. [24]

Meta-analysis	Iftikhar et al.
1. PICO components clearly defined	Yes
2. Predefined protocol and justification of deviations	Yes
3. Explanation of study design selection	Yes
4. Comprehensive literature search	Yes
5. Study selection in duplicate	Yes
6. Data extraction in duplicate	Yes
7. List and justification of excluded studies	No
8. Detailed description of included studies	Yes
9. Risk of bias assessment	Yes
10. Reporting of funding sources	Not specified
11. Appropriate meta-analysis methods	Yes
12. Impact of risk of bias on results	Yes
13. Consideration of bias in discussion	Yes
14. Explanation of heterogeneity	Yes
15. Investigation of publication bias	Limited (fewer than 10 studies)
16. Conflict of interest disclosure	Yes

Results

A total of 108 papers from PubMed were first screened for the literature search; this number was then lowered to 15 articles. The Cochrane database yielded eight articles during the initial screening, with only one article remaining after further evaluation. Google Scholar's first 10 pages were screened and contributed an additional seven articles to the pool. The final analysis included a diverse range of article types, comprising 16 review articles, one RCT, one open-label trial, one meta-analysis, and four observational studies. Ultimately, the comprehensive literature review incorporated a total of 23 articles, with 15 sourced from PubMed, one from Cochrane, and seven from Google Scholar.

**Figure 1:**
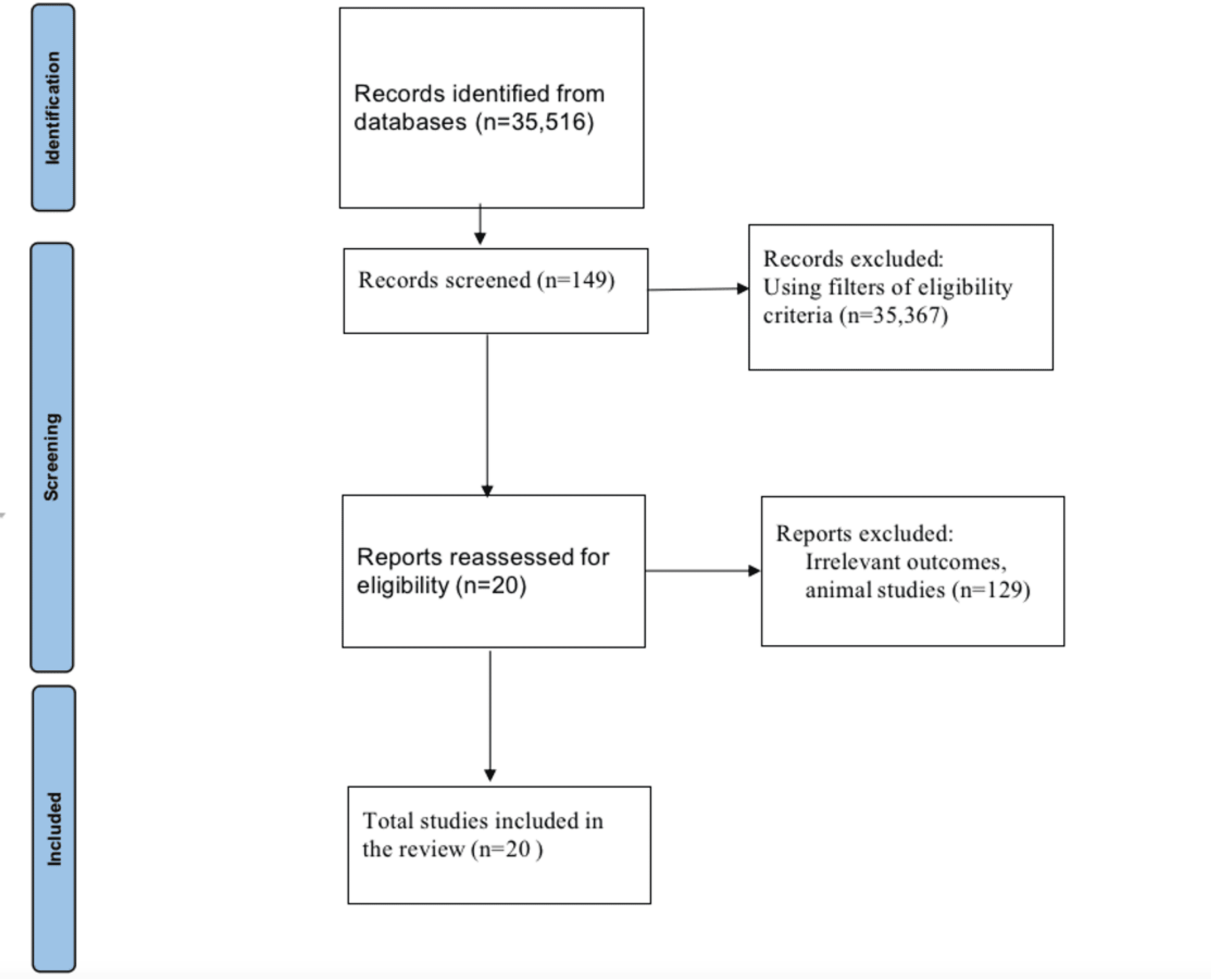
PRISMA flowchart PRISMA: Preferred Reporting Items for Systematic Reviews and Meta-Analyses

A group of 764 PD patients and 132 healthy controls were included in the study. Among the Parkinson's group, males were more prevalent, comprising 59.27% (358) of the patients, with an average age ranging from 64±3 years. The control group, in comparison, had a slightly lower male representation at 54.54% (72) and a mean age of 57 years. This demographic distribution aligns with global trends, which indicate a higher prevalence of PD among older adults and men. The patient and control groups exhibited similar body mass index (BMI), with average BMI values falling between 26 and 27. The PRISMA flowchart is displayed in Figure 1. The key findings of the included studies are displayed in Table 1.

Discussion

Altered Gut Microbiome and PD

Gut dysbiosis in PD is characterized by a complex microbial imbalance that potentially exacerbates the condition through the gut-brain axis. Huang et al. concluded that beneficial bacteria like *Faecalibacterium*, *Prevotella*, *Lachnospiraceae*, *Blautia*, *Coprococcus*, and *Roseburia*, which produce SCFAs, are typically depleted in PD patients. This depletion correlates with cognitive impairment, gait disorders, worse motor scores, and postural instability. The same study noted consistent enrichment of *Lactobacillus*, *Akkermansia*, and *Bifidobacterium* genera in PD patients [2].

Huang et al. also found that pro-inflammatory species such as *Escherichia*/*Shigella*, *Klebsiella*, and other *Enterobacteriaceae* are often increased in PD patients, correlating with disease duration and severity in cohorts ranging from 34 to 197 individuals. They noted that *Akkermansia muciniphila*, despite producing SCFAs, may contribute to gut barrier damage and alpha-synuclein aggregation when overabundant. The review highlighted that opportunistic pathogens like *Porphyromonas* and *Corynebacterium* are also enriched in PD fecal samples from studies involving 10-72 PD patients. *Enterococcus* species impact drug metabolism by decarboxylating levodopa to dopamine in the gut, potentially reducing its efficacy in PD patients. This overall shift in gut microbiota leads to a pro-inflammatory environment, increased gut permeability, and altered protein degradation pathways, which may exacerbate PD pathology [3].

Huang et al. conducted a study that used sequencing of 16S rRNA to compare the gut microbiomes of 29 PD patients with 29 age-matched controls. This study found no differences in alpha diversity (which measures the variety of species within a single sample). However, beta diversity analysis (which compares the diversity between different samples or groups) revealed significant differences in four bacterial families, with *Lactobacillaceae*, *Barnesiellaceae*, and *Enterococcaceae* greater in number in PD cases.

Çamcı and Oğuz reviewed multiple studies investigating the relationship between *Helicobacter pylori* (HP) infection as well as PD, encompassing a total of 493 PD patients, of which 182 had HP infection, according to different studies. HP-positive PD patients generally exhibited worse motor function, longer onset time, shorter "on-time" for levodopa treatment, and improved levodopa absorption and efficacy after HP eradication. They also had worse Unified Parkinson's Disease Rating Scale (UPDRS) and Parkinson's Disease Questionnaire 39 (PDQ39) scores, which improved following HP eradication and typically required higher levodopa doses. In vitro studies demonstrated that HP could decrease levodopa concentration in laboratory settings. However, Çamcı and Oğuz noted that one research by Narożańska et al. found no significant difference in UPDRS and Hoehn-Yahr scores between HP-positive and HP-negative patients, highlighting the complex nature of this relationship [4].

Huang et al. detailed a thorough investigation conducted by Keshavarzian et al. that examined 65 fecal samples and 66 sigmoid mucosal biopsies from 38 PD patients and 34 healthy controls. Keshavarzian et al. discovered significant variations in the microbial communities between PD patients and controls, particularly in fecal samples. In fecal and mucosal samples, healthy individuals showed higher levels of anti-inflammatory bacteria (*Blautia*, *Coprococcus*, *Roseburia*, and *Faecalibacterium*). In contrast, PD patients had more potentially pro-inflammatory bacteria (*Proteobacteria*) in their gut mucosa. These findings suggest a possible connection between gut microbiome composition and PD [5]. The summary of the altered gut microbiome in PD is given in Table 9.

**Table 9:** Summary of gut dysbiosis in PD PD: Parkinson's disease; SCFAs: short-chain fatty acids; LPS: lipopolysaccharides

Bacteria	Increase/decrease	Impact on gut microbiome	Mechanism of action relating to PD
Faecalibacterium	Decrease	Reduced the production of SCFAs, particularly butyrate	Decreased anti-inflammatory effects and gut barrier integrity, leading to increased neuroinflammation
Prevotella	Decrease	Reduced mucin production and SCFA levels	Impaired gut barrier function and increased gut permeability, contributing to systemic inflammation
Akkermansia	Increase	Increased mucin degradation	Contribute to gut permeability and inflammation in PD
Lactobacillus	Increase	Increased the production of lactic acid	May have dual roles: beneficial in gut health but overgrowth linked to dysbiosis and inflammation in PD
Bifidobacterium	Increase	Enhanced carbohydrate fermentation and SCFA production	Overgrowth may be associated with dysbiosis and altered immune responses in PD
Escherichia	Increase	Increased the production of LPS	LPS can trigger systemic inflammation and neuroinflammation, exacerbating PD pathology
Proteobacteria	Increase	Increased the presence of opportunistic pathogens	Associated with pro-inflammatory states and increased gut permeability, contributing to neuroinflammation
Bacteroides	Increase	Enhanced carbohydrate metabolism and SCFA production	Overgrowth linked to elevated inflammatory markers and gut permeability, contributing to PD progression
Roseburia	Decrease	Reduced butyrate production	Lower anti-inflammatory effects and compromised gut barrier, leading to increased neuroinflammation
Coprococcus	Decrease	Reduced SCFA production	Decreased anti-inflammatory effects, contributing to systemic and neuroinflammation in PD

Potential mechanisms linking gut microbiome to PD pathogenesis

Inflammation

Inflammation plays an important part in PD disease, as microbiota present in the gut make an impact on inflammation. "Leaky gut" is nothing but a condition of increased inflammation in the intestine and permeability in patients. TNF-α, IL-1β, and IL-6, which are pro-inflammatory cytokines, are present in high concentrations in the blood of the individual suffering from PD disease, suggesting the possibility of a connection with the disease. Scientists have also found that intestinal glial cells are activated and the level of expression of toll-like receptors is high in the colons of PD patients. This indicates that the state of inflammation is getting worse [6].

To check the strength of the gut wall of PD patients, many markers were used. A lot of the protease inhibitor alpha-1-antitrypsin is found in the feces of people with PD, so this means that the mucosal barrier is not strong and was unable to hold proteins into the intestinal lumen; high levels of zonulin were also present, which is a protein that is linked with junctions that are tight, in comparison to those of controls. Lactoferrin is found at a small level but still acts as a fecal indicator for inflammation in PD patients; calprotectin also performs as another indicator [7].

Calprotectin, alpha-1-antitrypsin, and zonulin levels were higher in the feces of 34 PD people compared to 28 age-matched controls that were found by Schwiertz et al. (2018) [7]. Forsyth et al. did another study with 10 healthy controls and nine people with PD. They found that the 24-hour urine sucralose level was higher in PD patients. Furthermore, the "leaky gut" hypothesis was further substantiated by the increased presence of *E. coli *in the lamina propria and epithelium of PD patients when compared to healthy controls, as evidenced by sigmoid staining with polyclonal antibodies to *E. coli *[8].

Researchers have looked at the tight junction proteins in intestinal biopsies from PD patients and found that there is less zonulin, an unusual subcellular distribution of tight junction proteins, and a lot less occludin. Overall, these results show that PD patients have intestinal inflammation and higher permeability, which may help the disease get worse by encouraging alpha-synuclein aggregation and brain inflammation [7].

Pathology of Alpha-Synuclein

Alpha-synuclein is a protein made up of 140 amino acids, and it is encoded by the SNCA gene. It consists of three distinct regions: an amphipathic N-terminal domain that associates with phospholipid membranes, a hydrophobic central section, and an acidic C-terminal region. Alpha-synuclein can exist in three different conformations: fibrillar, calcified, and monomeric. More frequently, the fibrillar form of the protein is connected with neurological and clinical disorders including PD [9].

The genetic influence of alpha-synuclein in the development of PD is complex. Mutations in the alpha-synuclein (SNCA) and LRRK2 genes are linked to autosomal dominant forms of PD, while mutations in genes like Parkin, PINK1, DJ-1, and ATP13A2 are associated with autosomal recessive forms. Gut dysbiosis, characterized by changes in the composition of the microbiota, produces a noticeable impact on the development of PD. Specific changes include an increase in possibly pathogenic and toxic bacteria such as *Enterobacteriaceae* and a decrease in health-promoting bacteria such as *Prevotellaceae*. Furthermore, one of the alterations concomitant with aging is an increase in the permeability of the small intestinal epithelium and a decrease in bacterial species that produce SCFAs which in turn increases the proportions of the most important bacterial products as well as uptake and intracellular transport of bacterial components including lipopolysaccharide (LPS) into the circulatory system [10].

Alpha-synuclein accumulation and neuroinflammation can be influenced by the altered gastrointestinal microbiome through a variety of mechanisms. The permeability of the blood-brain barrier can be influenced by the production of metabolites such as SCFAs, while bacterial products like LPS can induce alpha-synuclein accumulation and neuroinflammation. Furthermore, the immune system's activation may exacerbate neuronal injury by increasing the production of pro-inflammatory cytokines.

In physiological circumstances, alpha-synuclein has an important role to play like the release of neurotransmitters along with facilitating its transport, release, and reuptaking with the help of synaptic vesicle fusion complexes' interactions. The growth and maintenance of dopaminergic neurons, synaptic plasticity, and the equilibrium of neurotransmitter release depend on the protein. According to recent research, alpha-synuclein may play a messenger role in innate immunity, alerting immune cells in the central nervous system to the existence of specific pathogens.

In pathological conditions, alpha-synuclein undergoes misfolding and abnormal aggregation, leading to several neurodegenerative processes. The aggregation process involves transitioning from its standard soluble form to an aggregated beta-sheet conformation, culminating in the formation of Lewy bodies. Cytoplasmic structural proteins, complement proteins, ubiquitin, and misfolded alpha-synuclein comprise these neuronal inclusion bodies. Aggregated alpha-synuclein can cause significant neuronal dysfunction by disrupting the formation of SNARE complexes, essential for vesicle docking and fusion, and inducing mitochondrial dysfunction and oxidative stress. Additionally, these aggregates stimulate inflammatory responses in microglia, contributing to neurodegeneration and the eventual death of dopaminergic neurons in areas like the substantia nigra pars compacta, resulting in PD's characteristic motor symptoms.

Propagation of alpha-synuclein pathology is believed to occur through a prion-like mechanism. According to the gut-to-brain theory, the enteric nervous system (ENS) may be the site of alpha-synuclein aggregation's origin before it spreads to the brain. The vagus nerve allows misfolded alpha-synuclein from the stomach to the brain. Evidence supporting this includes studies showing that vagotomy could lower the danger of PD. Other potential propagation routes include endocrine-like transmission through the bloodstream, facilitated by increased gut and blood-brain barrier permeability, and the direct effects of bacterial metabolites on brain function [9].

Once in the brain, pathological alpha-synuclein can induce the misfolding of normal alpha-synuclein in a template-dependent manner, promoting the spread of pathology. This neuroinflammation is accompanied by astrocytes and microglia activation, leading to chronic inflammation that further contributes to disease progression [9].

SCFAs and Butyrate

The study conducted by Aho et al. offers convincing proof for the significant role of SCFAs, particularly butyrate, in PD pathophysiology. This comprehensive analysis involved 55 PD patients and 56 healthy controls, focusing on the intricate relationships among gut microbiota, SCFAs, inflammation, and intestinal permeability [11].

Aho et al. found that stool samples from PD patients had significantly lower levels of SCFA, particularly butyrate, compared to healthy controls. This decrease was linked to changes in the makeup of the gut microbiota, indicating a critical role of gut health in the initiation and development of PD. In line with the lower amounts of SCFA, the research found that PD patients had greater levels of fecal calprotectin, a marker of increased intestinal inflammation.

Butyrate emerged as a critical player in PD pathophysiology, with several essential functions. It helps to improve the expression of tight junction proteins, which is important for keeping the gut barrier working properly. Lower butyrate levels in PD may lead to increased gut permeability, potentially allowing bacterial products to enter systemic circulation and trigger inflammatory responses. Neuroprotective butyrate influences neurotransmitter balance, particularly enhancing gamma-aminobutyric acid (GABA) synthesis and release. This modulation may help regulate the excitation-inhibition balance often disrupted in PD.

As a histone deacetylase (HDAC) inhibitor, butyrate impacts gene expression related to PD pathology, potentially altering alpha-synuclein accumulation and enhancing neurotrophic factor expression for dopaminergic neuron survival. By blocking pro-inflammatory pathways like nuclear factor kappa B (NF-κB) and stimulating anti-inflammatory processes in the gut, SCFAs, particularly butyrate, have potent anti-inflammatory effects. Furthermore, butyrate activates the Nrf2 pathway, promoting the expression of antioxidant enzymes that protect against oxidative stress implicated in PD pathogenesis. Additionally, it boosts mitochondrial biogenesis and provides energy to colon cells, assisting in preserving cellular energy balance [12].

The results of Aho et al. emphasize how crucial the microbiota-gut-brain axis is in PD. SCFAs serve as crucial mediators in this pathway, influencing central nervous system function by modulating the ENS and engaging in systemic effects related to inflammation and metabolism. The study revealed that gut microbial interactions with the host may be altered in PD compared to healthy individuals, suggesting potential avenues for therapeutic interventions.

Interestingly, Aho et al. found that higher butyrate levels correlated with the later onset of both motor and non-motor symptoms in PD patients, indicating a possible protective effect. The study also highlighted sex-dependent differences, with butyrate reductions being more significant in male PD patients, while inflammatory marker variations differed across genders [11].

Tryptophan and Serotonin (5-HT) Metabolism

Tryptophan is primarily obtained from the diet and is metabolized by gut bacteria, such as *E. coli*, into ligands for the aromatic hydrocarbon receptor via the kynurenine (KP) and 5-HT pathways. The KP produces both neuroprotective metabolites, such as kynurenic acid (KYNA), and neurotoxic metabolites, including quinolinic acid (QA) and 3-hydroxykynurenine (3-HK). In PD patients, tryptophan metabolism often favors neurotoxic pathways. KYNA levels are reduced in regions like the putamen, substantia nigra pars compacta (SNpc), and frontal cortex, while QA in plasma and 3-HK in the SNpc and putamen are elevated. These metabolites may inhibit mitochondrial complex I, leading to increased neurotoxicity and oxidative stress, further damaging mitochondria and resulting in the loss of dopaminergic neurons in the SNpc [22].

Tryptophan also regulates 5-HT synthesis in the central nervous system, influencing behavior, emotion, and memory. The severity of resting tremor and cognitive decline in PD correlates with 5-HT neuron degeneration. Melatonin, a 5-HT pathway metabolite, may alleviate non-motor symptoms of PD by reducing oxidative stress and inhibiting mitochondrial-dependent apoptotic pathways. It also enhances mitochondrial bioenergetics by supplying acetyl-CoA, optimizing mitochondrial function. Modulating tryptophan intake could offer therapeutic benefits for PD [22].

A meta-analysis of five RCTs with 155 patients showed significant reductions in the UPDRS total scores for those receiving melatonin at 10 mg/day or higher doses. The mean difference (MD) was -11.35 with a 95% confidence interval (CI) from -22.35 to -0.35, and the heterogeneity was I²=0% and p=0.04. These improvements were observed with immediate-release formulations. No significant effects were found on individual UPDRS II, III, and IV scores, regardless of dosage or duration. However, significant improvements in Pittsburgh Sleep Quality Index (PSQI) scores were noted with immediate-release melatonin formulations (MD=-2.86, 95% CI: -4.74 to -0.97, I²=0%, p=0.003), indicating enhanced sleep quality [24].

Bile Acids (BAs)

BAs such as chenodeoxycholic acid (CDCA) and cholic acid are synthesized in the liver, stored in the gallbladder, and released into the duodenum after eating. While most are reabsorbed in the intestine, some reach the colon, where bacteria like *Clostridium* convert them into secondary BAs. In PD patients, ursodeoxycholic acid (UDCA), a minor BA component, is found in a reduced glycine-bound form in plasma. In contrast, primary liver-derived and secondary BAs are elevated. Levodopa treatment can help mitigate these elevations [22].

UDCA and tauro ursodeoxycholic acid (TUDCA) have shown benefits in PD models by enhancing motor performance, improving mitochondrial function, and reducing neuroinflammation. TUDCA acts as an anti-apoptotic agent by promoting mitophagy through PINK1 and Parkin expression, clearing damaged mitochondria, and supporting neuronal survival [22].

An open-label study with five PD patients evaluated oral UDCA at increasing doses over six weeks. Measurements included blood safety panels, plasma concentrations of UDCA (via liquid chromatography-mass spectrometry), and brain ATP levels (via 7-Tesla 31P magnetic resonance spectroscopy). Secondary assessments involved the UPDRS and Montreal Cognitive Assessment (MOCA). UDCA was generally well tolerated, with mild to moderate gastrointestinal discomfort as the most common adverse event. Pharmacokinetic analysis showed a maximum concentration of 8749±2840 ng/mL and a half-life of 2.1±0.71 hours. Magnetic resonance spectroscopy data indicated modest increases in ATP levels and decreases in ATPase activity, with clinical score changes of -4.6±6.4 for the UPDRS and 2±1.7 for the MOCA [23].

Microbial gut-brain axis

Studies by Omotosho et al. and Menozzi et al. described the "gut-brain" theory for PD etiology, proposed by Braak and colleagues in 2003, suggesting that an unidentified neurotropic pathogen may enter the gastrointestinal tract, causing pathological alpha-synuclein aggregation in the ENS. This aggregated alpha-synuclein could travel through the vagus nerve to reach the brain, potentially contributing to PD development. Evidence supporting this theory includes the discovery of Lewy bodies in the ENS and dorsal motor nucleus of the vagus (DMV) of PD patients, as well as alpha-synuclein immunoreactive inclusions in the submucosal Meissner plexus and myenteric Auerbach plexus of patients with Lewy body-related brain disease.

A comprehensive Danish study described by Fan et al. examined the relationship between vagotomy procedures and PD risk. After analyzing 11,209 patients over a 20-year follow-up period, the patients were divided into two groups: 5,339 individuals who underwent complete vagotomy and 5,870 who received highly selective vagotomy. The study revealed that patients who had undergone truncal vagotomy demonstrated a lower incidence of PD compared to those who had a super selective procedure. This finding supports the vagus nerve's role in PD pathogenesis [13]. Additionally, Fitzgerald et al. reported that animal studies have shown vagal efferent axons and terminals expressing alpha-synuclein and that vagotomy can partially inhibit the development of motor impairment and the spread of pathology in PD animal models [9].

Large population-based studies suggest that vagotomy, particularly truncal vagotomy, reduces the risk of developing PD more than five years after the procedure. The vermiform appendix has been proposed as a possible initial site for enteric alpha-synuclein aggregation due to its high alpha-synuclein concentration, lack of blood-tissue barrier in the mucosa, and connection to vagal afferents. Interestingly, studies have shown that appendectomy both lowered the chance of developing PD and delayed its onset, further supporting the gut-brain connection in PD pathogenesis [9].

Over the past two decades, researchers have been evaluating alpha-synuclein in the gastrointestinal tract of PD patients during manifest and prodromal phases, as noted by Liu et al. However, the findings have been contradictory. Phosphorylated alpha-synuclein deposition, a prodromal hallmark of PD, has been observed in submucosal nerve fibers or ganglia of patients with idiopathic rapid eye movement sleep behavior disorder (iRBD). Similar results were found in the ENS of stomach, duodenal, and colonic biopsies from PD patients years before motor symptom onset, suggesting pathogenetic alterations at the prodromal stage [12]. However, Menozzi et al. pointed out that isolated alpha-synuclein pathology in the gastrointestinal system remains relatively rare [6]. Some studies found similar alpha-synuclein or phosphorylated alpha-synuclein immunoreactivity in intestinal samples from PD patients and healthy age-matched controls, although quantitative morphometry revealed differences in the amount and pattern of phosphorylated alpha-synuclein aggregates between groups [12].

Several factors contribute to the variability in pathological findings, including a lack of standardized immunohistochemical protocols, differences in gastrointestinal areas investigated, and extensive dispersion of peripheral nerve branches. To address these disparities, new functional imaging techniques offer non-invasive evidence of the gut-brain axis's importance in vivo. The PET tracer [11C]-donepezil can measure acetylcholinesterase density in peripheral organs, indicating parasympathetic gut innervation. Recent research has strengthened the case for a "gut-first" subtype of PD. Studies found that putaminal 18F-dihydroxyphenylalanine (FDOPA) PET uptake was similarly reduced in PD patients with prodromal RBD.

De novo PD patients with prodromal RBD showed reduced 123I-metaiodobenzylguanidine (MIBG) heart-to-mediastinum ratios, reduced colon [11C]-donepezil uptake values, enlarged colon volumes, and delayed colonic transit times. These findings collectively support the hypothesis of a gut-brain connection in PD development, particularly in a subset of patients. However, further research is needed to fully understand the implications of these observations and their potential for the early diagnosis or treatment of PD [12].

Therapeutic implications

Treatment strategies that focus on the gut microbiota have demonstrated encouraging outcomes in controlling PD symptoms and maybe slowing the disease's progression. Probiotics have been shown in several clinical trials to aid patients with PD. Over the course of four weeks, Barichella et al. discovered that fermented milk containing several probiotic strains helped PD patients' constipation symptoms [15]. A probiotic mixture combining *Lactobacillus* and *Bifidobacterium* strains decreased Modified Unified Parkinson's Disease Rating Scale (MDS-UPDRS) total scores after 12 weeks of treatment, according to a different study by Tamtaji et al. [14]. Sun et al. found that after three months of *Bifidobacterium animalis* subsp. *lactis* Probio-M8 medication, there were improvements in motor symptoms, sleep quality, constipation, and cognition [25]. These results imply that probiotics may assist in reestablishing good bacteria, enhancing the integrity of the intestinal barrier, and lowering inflammation in PD patients [15].

While probiotics have been more extensively studied, prebiotics have also shown potential in PD management. Becker et al. conducted an open-label trial and found that prebiotic-resistant starch supplementation improved non-motor symptoms and reduced fecal inflammatory markers in PD patients [26]. Prebiotics could potentially stimulate the growth of beneficial bacteria and improve gut barrier function.

It has been shown that dietary changes may alter the gut microbiota in PD. In research with 54 PD patients and 34 healthy controls, Hegelmaier et al. showed that after a year, PD patients who additionally received fecal enemas experienced improvements in their motor scores and reduced their pharmaceutical demands. The role of SCFAs produced by gut bacteria in modulating PD pathogenesis has been highlighted, suggesting that dietary interventions to promote SCFA production by beneficial bacteria may be a therapeutic strategy. Interestingly, epidemiological studies have found a lower incidence of PD in smokers and coffee drinkers, presumably due to microbiome alterations that enhance gut barrier function and lower inflammation [16].

Targeting specific bacteria has been proposed as another therapeutic approach. Some studies have shown that eradicating *H. pylori *infection in PD patients improves levodopa absorption and motor symptoms, while others have found no benefit. Inhibiting *Enterococcus faecalis*, which can decarboxylate levodopa, may improve the drug's bioavailability. Reducing the abundance of pro-inflammatory *Desulfovibrio *bacteria, which may promote alpha-synuclein aggregation, could potentially slow PD progression [17].

FMT

The research done by Sun et al. and Cheng et al. provides interesting new information about the possibility of FMT as an adjunctive treatment for PD [25,18]. Cheng et al.'s randomized, placebo-controlled trial involving 56 PD patients with 27 patients in each group completing the trial demonstrated significant improvements in PD-related autonomic symptoms in the FMT group compared to placebo at 12 weeks, as measured by the MDS-UPDRS total score (group×time effect, B=-6.56 (-12.98, -0.13), p<0.05) [18].

Sun et al.'s animal model study showed that FMT rebalances the gut microbiome, explicitly increasing beneficial bacteria like *Firmicutes* and *Clostridiales* while reducing potentially harmful ones such as *Proteobacteria*, *Turicibacterales*, and *Enterobacteriales* [25]. FMT treatment in PD mice reduced fecal SCFA, improved physical function, and enhanced 5-HT and dopamine neurotransmitter levels in the striatal pathways. FMT exhibited neuroprotective properties in mouse models of PD through multiple mechanisms. In the substantia nigra, FMT reduced astrocyte and microglia activation. Additionally, it attenuated the TLR4/TNF-α signaling cascade in both cerebral and intestinal tissues. These effects collectively contribute to FMT's potential as a therapeutic approach for PD by modulating neuroinflammation and gut-brain interactions [19].

Both studies highlighted the positive effect of FMT on gastrointestinal disorders associated with PD. Cheng et al. reported significant improvements in Irritable Bowel Syndrome Quality of Life (IBS-QOL) score, Gastrointestinal Symptom Rating Scale (GSRS) score, and IBS-Symptom Severity Score (IBS-SSS) in the FMT group, suggesting an improved quality of life in stomach pain and flatulence. Improvements in cognitive function were also observed among FMT recipients, as measured by the Mini-Mental State Examination (MMSE) and MOCA scales.

Comparative studies of gut microbiota between healthy individuals and PD patients revealed differences. Cheng et al. noted reduced abundances of beneficial bacteria such as *Blautia*, *Coprococcus*, and *Roseburia* in PD patients. Post-FMT treatment showed increased beta diversity in the microbiome with notable rises in *Firmicutes* families and genera like *Blautia*, *Roseburia*, and *Faecalibacterium* but a decline in *Bacteroides*. These changes persisted for at least three months post-treatment, correlating with decreased constipation and improved gut motility. Cheng et al. identified subgroups of FMT responders (FMT.R) and non-responders (FMT.NR), emphasizing further research to understand factors affecting FMT efficacy. Both studies reported good acceptance of FMT with minor adverse events [18].

Xue et al.'s small-scale study with 15 PD patients explored FMT delivery methods: colonoscopy for 10 participants and nasal-jejunal tube for five. Colonoscopic FMT showed more favorable outcomes, including improved motor function (UPDRS III) and sleep quality and partial alleviation of anxiety and depression symptoms after three months. However, five participants reported gastrointestinal side effects like loose stools and flatulence [20].

In a study by Zhong et al., a 1-methyl-4-phenyl-1,2,3,6-tetrahydropyridine (MPTP)-induced PD mouse model demonstrated that FMT improved motor symptoms, reduced fecal SCFAs, and decreased alpha-synuclein expression in the substantia nigra pars compacta (SN), as measured by Western blot. Further prospective human trials are necessary to confirm these findings in humans [27].

Dietary intervention

The article by Jackson et al. gives a detailed overview of how diet can shape the gut microbiome and its potential impact on the development and progression of PD. The authors discuss several key dietary factors that affect the gut microbiome in the context of PD.

The Western diet, high in refined sugars and low in fiber and saturated fats, has been linked to a higher risk of PD. According to Jackson et al., this type of diet can lead to an increase in pro-inflammatory bacteria in the gut and a decrease in helpful bacteria. This imbalance can result in increased intestinal permeability and systemic inflammation, potentially contributing to PD development. On the other hand, a lower risk of PD has been associated with a Mediterranean diet that includes high amounts of fruits, vegetables, whole grains, and omega-3 fatty acids. This diet can enhance the growth of beneficial bacteria, which produce SCFAs. SCFAs have anti-inflammatory and potential neuroprotective effects [20].

High fiber intake was connected to a reduced risk of PD. Jackson et al. explain that dietary fiber is a prebiotic, promoting the growth of SCFA-producing bacteria like *Bifidobacterium *and *Lactobacillus*. These bacteria help maintain gut barrier integrity and reduce inflammation. The consumption of probiotics and fermented foods may also positively influence the gut microbiome in PD patients. The authors mention that these foods can increase beneficial bacteria and potentially alleviate some PD symptoms, though more research is needed. Additionally, dietary polyphenols found in fruits, vegetables, and tea have been shown to modulate the gut microbiome. Jackson et al. note that these compounds may have neuroprotective effects and could potentially slow PD progression [21].

The article also discusses the role of diet-induced microbiome changes in PD. Increased intestinal permeability brought on by diet-induced dysbiosis can allow bacterial toxins like LPS to enter the circulation. Jackson et al. explain that this "leaky gut" phenomenon may contribute to systemic inflammation and neuroinflammation in PD. Certain gut bacteria can produce neurotoxic metabolites, and the authors highlight that an imbalanced microbiome may lead to increased production of these harmful compounds, potentially contributing to neurodegeneration in PD.

The gut microbiome may influence the pathology of PD in terms of alpha-synuclein aggregation. Jackson et al. suggest that certain bacterial species may promote or inhibit this aggregation, potentially affecting PD progression. Diet-induced changes in the gut microbiome can also affect the ENS. The authors note that this may contribute to gastrointestinal symptoms commonly seen in PD patients and might contribute to the onset of a disease and progression [21].

Lastly, the immune system's regulation is greatly influenced by the gut microbiota. According to Jackson et al., diet-induced dysbiosis might result in long-term, low-grade inflammation, which may exacerbate neuroinflammation and neurodegeneration in PD. This comprehensive review underscores the complex interplay between diet, gut microbiome, and PD, highlighting the potential for dietary interventions as a promising avenue for PD prevention and management. However, the authors also emphasize the need for further research to fully elucidate these relationships and translate these findings into clinical practice [21].

Limitations and challenges

Research into the gut microbiome's influence on PD faces several hurdles. Establishing causality between microbial alterations and PD development is challenging, as most studies are observational in nature. To address this, researchers need to conduct more longitudinal studies and develop experimental models. The field also grapples with methodological inconsistencies, as variations in sample handling, sequencing approaches, and data analysis can lead to conflicting results. Standardizing protocols could significantly improve study reproducibility and comparability. Additionally, translating research findings into effective clinical treatments remains a complex task, with issues surrounding treatment personalization and long-term safety yet to be fully resolved. It's important to note that these observations are based on recent human studies published in English, which also limits the available data.

## Conclusions

Growing evidence suggests a complex relationship between gut microbiota and PD development and progression. Studies have documented differences in gut microbiota composition between individuals with PD and healthy controls, including alterations in the levels of pro-inflammatory bacteria and those that produce SCFAs and anti-inflammatory compounds. These microbial changes may influence PD through various mechanisms, including altered intestinal permeability, neuroinflammation, and potential effects on alpha-synuclein aggregation and propagation via the gut-brain axis. This systematic review is essential as it consolidates current evidence suggesting a possible link between gut microbiota alterations and PD pathogenesis. The review highlights the possibility that therapeutic strategies targeting the gut microbiome may complement existing treatments and improve the management of both motor and non-motor symptoms.

The results suggest that treatments targeting the gut microbiome, such as probiotics, prebiotics, synbiotics, and FMT, may potentially alleviate both motor and non-motor symptoms. Additionally, understanding the gut-brain axis could pave the way for early diagnostic markers and preventive strategies. Future research should focus on conducting large-scale, longitudinal studies to establish causality, standardizing microbiome analysis methods, evaluating FMT's impact on alpha-synuclein in humans, examining the relationship between PD medications and gut microbiome, developing personalized microbiome-based therapies, and identifying specific microbial markers for early diagnosis or preventive interventions.
